# Development and deployment of a solid oral amphotericin B dosage form to treat visceral leishmaniasis within a pediatric population

**DOI:** 10.1371/journal.pntd.0012500

**Published:** 2024-09-26

**Authors:** Kishor M. Wasan

**Affiliations:** 1 Department of Urologic Sciences, Faculty of Medicine, University of British Columbia, Vancouver, British Columbia, Canada; 2 The Neglected Global Diseases Initiative, University of British Columbia, Vancouver, British Columbia, Canada; Institute of Medical Sciences, INDIA

## Abstract

Visceral leishmaniasis (VL) is a severe and potentially fatal infection, with over 90% of reported cases occurring in East African countries including Chad, Djibouti, Eritrea, Ethiopia, Kenya, Somalia, South Sudan, Sudan, and Uganda, affecting mainly impoverished individuals, and creating a significant economic burden. Currently, the intravenous single-dose liposomal amphotericin B is the first choice for the treatment of VL. Recently, WHO and DNDi have suggested a combination of intravenous liposomal amphotericin B and oral miltefosine as a potential approach to treat VL. However, miltefosine availability is uncertain, and its side effects frequently cause treatment to be discontinued. Furthermore, due to the difficult route of liposomal amphotericin B administration by intravenous infusion, the lack of formulation’s tropical stability, accessibility, injection toxicity, and cost have prevented this injectable formulation of amphotericin B from reaching the most infected populations, particularly the pediatric population. To solve this problem, the development of a solid oral amphotericin B formulation that is cost-effective, safe, tropically stable, and easy to swallow, making it more accessible to children, particularly in rural communities having limited access to medical clinics or trained healthcare professionals is imperative. This viewpoint will discuss the opportunities and challenges of developing an oral amphotericin B formulation for a pediatric population.

## Introduction

Visceral leishmaniasis (VL) is a severe and potentially fatal infection, with over 90% of reported cases occurring in Eastern African countries—Chad, Djibouti, Eritrea, Ethiopia, Kenya, Somalia, South Sudan, Sudan, and Uganda, affecting mainly impoverished individuals, and creating a significant economic burden through direct and indirect treatment costs. Recently, WHO and DNDi have suggested a strategy to treat VL with a combination of intravenous liposomal amphotericin B and oral miltefosine [1].

However, miltefosine availability remains uncertain in both VL and PKDL populations, and its side effects frequently cause treatment to be discontinued. Furthermore, due to the difficult route of liposomal amphotericin B administration by intravenous infusion requiring medical infrastructure and trained medical personnel, the lack of formulation’s tropical stability, accessibility, injection toxicity [2–4] and cost have prevented this injectable amphotericin B formulation from reaching the most infected populations.

To address this problem, several groups are developing oral amphotericin B formulations that are cost-effective, safe, tropically stable, and easy to administer, making it more accessible to patients, particularly in rural communities having limited access to medical clinics or trained healthcare professionals. This would add a new therapeutic drug formulation to the existing drug pipeline.

*As directly stated recently by Texas Children’s Hospital Global Health Team “Roughly 350 million people are at risk*, *living in areas endemic to leishmaniasis*. *The most recent estimates from the Global Burden of Disease Study (GBD) 2015 indicate that an average of more than 24*,*202 deaths result annually from Leishmaniasis and 3*.*3 million disability-adjusted life years (DALYs) are accrued*, *ranking Leishmaniasis*, *along with schistosomiasis and hookworm infection*, *as those neglected tropical diseases (NTDs) with the highest disease burden*.*”* [2,3].

The current standard of care is not cost-effective from an accessibility point of view, cannot be stored in tropical temperatures, is administered as an injection (i.e., liposomal Amphotericin B and Paromomycin) which makes it inaccessible to millions in rural communities and has systemic and injection toxicities [4]. An oral amphotericin B product will address these limitations by being cost-effective, safe, tropically stable, and accessible to vulnerable communities (primarily women and children in rural communities).

## Development of a solid oral amphotericin B dosage form to treat VL

Extensive preclinical and clinical research has been completed on the development of several oral amphotericin B products by several research groups. This includes developing a tropically stable formulation with over 24 months of stability in developing world conditions (following ICH guidelines), testing this formulation in validated VL animal models for efficacy and toxicity and completing a human Phase 1a/1b safety study. Further to its activity against parasitic and fungal diseases, it has been shown that amphotericin B has immunomodulatory activity as well making it a strong candidate for immunocompromised patients that have these infections [5–10].

Colleagues at the University of British Columbia (UBC), Neglected Global Diseases Initiative group have also worked to solve the task of developing an oral AmB formulation for many years [5]. Their approach was to develop a lipid-based self-emulsifying drug delivery system (SEDDS) for AmB to permit oral administration of this poorly bioavailable drug with an additional aim of lessening its nephrotoxicity while maintaining optimal antileishmanial activity [7–10]. The authors employed mono- and di-glycerides in addition to D-alpha-tocopheryl poly (ethylene glycol) succinate (vitamin E–TPGS). An additional goal was to provide stability for AmB in their delivery system to withstand tropical temperatures, considering the clinical target. Before deciding on the iCo-010 formulation, which has recently completed Phase I human safety clinical trials, many versions of the formulation were developed and tested for stability, safety, and efficacy. iCo-010 was determined to be the most promising formulation with optimal stability (>75% over 60 days in 30°C; >95% after 4 h in simulated intestinal fluid) antileishmanial activity was observed in a murine model of VL, where <99% reduction in parasitic infection was achieved following 5 days of treatment with 10 mg/kg po twice a day (bid) and 95% inhibition following treatment with 20 mg/kg po qd (once a day) for 5 days, relative to the control. This formulation also exhibited more desirable self-emulsifying properties compared to other versions of the formulation, namely, iCo-011, -012, -013. The authors hypothesized that the desirable efficacy of their oral AmB formulation was likely a result of improved solubility, stability in the gastrointestinal tract, membrane permeability, and its ability to target the lymphatic transport system. The latter improvement may permit this formulation to target the greatest sites of infection in VL-infected organisms. Further investigation into the safety of the iCo-010 formulation found no evidence of GI toxicity, hepatotoxicity, or nephrotoxicity following the oral administration of multiple doses in a murine model. The biodistribution of the formulation in a mouse model showed uptake in the organs of the reticuloendothelial system at levels above the IC50 for the leishmania organism, which propelled iCo-010 into Phase I clinical trials. Furthermore, the potential use of iCo-010 for indications other than VL was explored, e.g., systemic candidiasis, which was found to be an effective once-daily 5-day treatment for this indication in a rat model.

Two human Phase I clinical studies have been recently completed [11]. In the Phase 1a human clinical study, the primary endpoint of safety and tolerability of our oral amphotericin B formulation following administration of all single ascending doses were met, including no signs of kidney, liver, and gastrointestinal (GI) toxicities of note. In addition, our oral amphotericin B formulation achieved a median plasma Cmax of 28 ng AmB/ml and AUC (0-inf) of 1,030 hr*ng/ml at the lowest dose of 100 mg. At the 400 mg dose, a median AUC (0-inf) of 2,029 hr*ng/ml was achieved, representing an approximate doubling of the AUC measure at an increased dose. In the Phase Ib human clinical study, all repeated doses of our oral amphotericin B formulation were well tolerated with no serious adverse events, including no signs of GI, kidney, and liver toxicities. Our oral amphotericin B formulation at the 100 mg dose achieved a median plasma Cmax of 26 ng AmB/ml and AUC (0-inf) 991 hr* ng/ml after day 1 of dosing and a median plasma Cmax of 44 ng AmB/ml and AUC (0-inf) 1,998 hr*ng/ml after 10 days of dosing. This approximate doubling of the AUC (0-inf) measure between day 1 and day 10 was observed not only at the 100 mg dose but at the 400 mg dose as well. See attached summary of the data in the other documents section.

Currently, there is only 1 oral amphotericin B formulation that has reached Phase II clinical studies in the treatment of vulvovaginal candidiasis (VVC) but not VL. This product from Matinas Biopharma (CAMB) did not show superior efficacy to the current standard of care for the treatment of VVC and displayed some gastrointestinal toxicity. However, the findings do suggest that their formulation shows enough systemic exposure to warrant further development in the treatment of VL. The UBC Oral AmB formulation has close to twice the systemic exposure of amphotericin B compared to the CAMB formulation* and the 100 mg UBC Oral AmB formulation has a higher systemic amphotericin B exposure than the 800 mg dose of CAMB**. The UBC Oral Formulation reports no adverse effects in its Phase 1a (single dose) or Phase 1b (multiple dose) studies. The CAMB formulation obtained a 55% clinical cure rate at 400 mg in VVC. Yet, the UBC formulation has twice the systemic amphotericin B exposure as the CAMB at the 400 mg dose with no adverse effects suggesting its clinical cure rate could be substantially higher than this formulation [6]. However, additional clinical studies are required to confirm this. In addition, a preliminary costing analysis was completed and using readily available and affordable excipients that have FDA GRAS approval [5] it was found treatment would be only $1 to 2 USD per day, making this a promising alternative to current injectable forms of the drug.

## Pediatric population challenges

Developing of any medication for the pediatric population is a unique challenge [12]. Dosing regimens in many diseases including leishmaniasis are traditionally tested primarily in adult populations and only later in children. Except for miltefosine, medications for the treatment of VL are administered parenterally: intramuscular injections are painful and often unsuited, given that many children with VL are severely malnourished, with no muscle mass; venous access for intravenous infusion is challenging—hence, the risk of nerve damage, thrombophlebitis of vein, and muscle abscess. Better treatment for children with VL can be achieved by combing short-term options—improved formulations and dosing regimens of existing medications—and longer-term research and development projects.

## Next steps

An oral formulation of amphotericin B that is cost-effective, safe, tropically stable, and easy to administer was also prioritized for development in the short term [13]. However, the desired characteristics (dosage form, strength) of this formulation are pending, awaiting the results of ongoing studies. These include a Phase II study exploring amphotericin B loaded in cochleates (to delay release in gastrointestinal media) for cryptococcal meningitis, and Phase Ia (single-dose), Ib (multiple-dose) studies to evaluate safety, tolerability, ideal systemic exposure, and pharmacokinetics/pharmacodynamics of novel lipid-based self-emulsifying oral amphotericin B formulations including syrup and capsule formulations that have already proved stable at tropical temperatures. However, it was noted that more data are needed to understand exposures with new oral formulations of amphotericin to ensure that exposure is optimized to achieve optimal outcomes. While new chemical entities are at different stages of development (some have reached early clinical phases), it will still take some time before children with VL will benefit from them. It is paramount that any effort to improve treatment take account of their final availability and affordability to ensure equitable access to the product where and when needed.

Recently, the WHO PADO working group completed a published report listing a solid dosage form of oral amphotericin B on their priority list (see Table 1; page 27 of the report; https://cdn.who.int/media/docs/default-source/campaigns-and-initiatives/gap-f/j0155_who-gapf_pado-for-neglected-tropical-diseases-meeting-report_v7.pdf?sfvrsn=7a18cce8_3 for development and deployment to children with VL within the next several years. With this endorsement from WHO, it is hoped oral amphotericin B development will be accelerated to help children in need.
